# Clinical Lecture on Chronic Albuminuria

**Published:** 1874-01-01

**Authors:** Austin Flint


### CLINICAL LECTURE ON CHRONIC ALBUMINURIA.

DELIVERED AT BELLEVUE HOSPITAL, N. Y., BY PROF AUSTIN FLINT, M.D

*From the New York Medical Record, December* 15, 1873.

GENTLEMEN: The topics which I shall present to you today embrace many features which are of much interest and importance, but which I shall be able to consider only in part. We have already considered acute desquamative nephritis, and now I wish to introduce for your consideration and study the different forms and manifestations of chronic disease of the kidneys. The existence of these affections is recognized by the changes which are manifested in the urine, and also by certain consequences resulting from renal disease. I wish to call your attention to certain points which will somewhat simplify and systematize your study; and I shall ask you to carefully read what has been written by some standard author or authors upon the different forms of chronic degenerative diseases of the kidneys, the effects which result from these different forms, and the circumstances which are involved in the differentiation, each from the others.

The most generally adopted classification of chronic diseases of the kidneys, or chronic Bright's disease, embraces four forms, namely: The large white kidney; the cirrhotic, or fibroid kidney; the fatty kidney, which some authors do not regard as a distinct form; and the amyloid, waxy, or lardaceous kidney. What effects do these different affections, severally and collectively, produce in the body?

These may be conveniently arranged in two classes: First, a diminished density of the blood, due to a constant elimination of albumen in the urine. This undoubtedly is an important element in the production of the dropsy which is so constantly present in these affections; but I would not be understood as saying that the loss of albumen, and consequent reduction in the density of the blood, is the sole cause of the dropsical manifestations.

The second class embraces effects which are due to the retention in the blood of excrementitious materials which should be eliminated from the system by the kidneys.

With the impoverished condition of the blood, which is in proportion to the loss of albumen, we have the dropsy, anæmia, and all those ulterior effects which arise from an anæmic condition; and, with the second class, we have all the effects which arise from the morbid conditions of the blood, caused by the retention of the excrementitious constituents of the urine.

The symptoms to which the latter of the two classes of effects give rise may be divided into the minor and grave symtoms. Among the minor symptoms are headache, nausea, and vomiting; looseness of the bowels, muscular cramps, etc. These are important symptoms, for the reason that they furnish evidence of a renal affection leading us to investigations which relate to the kidneys. More serious symptoms are those which denote inflammations, chiefly of the serous membranes, namely: pericarditis, pleuritis, and meningitis. Still graver symptoms are convulsions and coma.

With this brief outline, I shall bring before you cases illustrative of chronic renal disease.

The first case is a girl aged eighteen, a domestic. The countenance of this patient is quite typical. It is pallid, showing anæmia; and puffy, showing dropsy. There is a certain amount of anasarca present, not marked, but sufficient to show that the dropsy is diffused through the areolar tissue. A very reliable method of determining whether diffused dropsy is present or not, even in a very slight degree, is to make pressure over the sternum. If there be œdema, it can be recognized at that point. An important question to be decided now is, does the dropsy in the present case arise from an affection of the kidneys, or from an affection of the heart? It may be laid down as a general rule that, if there be much general dropsy, unaccompanied by difficulty in breathing, the dropsy can hardly arise from cardiac lesion. There is no evidence of heart disease in this case. Examination of the urine gives a s. g. 1018 acid; it contains considerable albumen, epithelial and granular casts and urates.

Let us now turn to the history of the case. Her family history is good. Patient is temperate; no evidence of specific disease. Two years ago—and this is a point of much interest—the patient had scarlet fever. It will be recollected that, while studying the acute form of Bright's disease, your attention was called to the fact that a great majority of the cases of acute albuminuria, or tubal nephritis, are cases in which the affection is a sequel of scarlet fever. It was also remarked that the acute affection rarely terminates in a chronic condition. But it seems probable that the case before us is a chronic affection, and that it dates its commencement from the occurrence of the scarlet fever; in other words, that we have here a chronic affection of the kidney following an acute tubal nephritis. Since she had the scarlet fever, her feet, face and body have occasionally become puffy, and the amount of urine passed has been sometimes quite scanty. Her face has never regained its natural color; and her strength has been very much diminished. She dates her present sickness at four days before her admission into the hospital. While in a profuse perspiration she sat down in a current of cold air, and she was seized with slight chill, with severe pain in the left side, and afterwards in the right side. Upon admission the pulse was frequent, the temperature raised, and the respirations rapid. To-day a physical examination of the chest reveals fluid in both pleural cavities. Now, a question of interest is, is this hydrothorax dependent upon the renal disease, or is it a case of double pleurisy? I do not hesitate to say that it is a case of double pleurisy. It is a case of double pleurisy which proceeds from renal disease, without much general dropsy. With but little general dropsy, and with no disease of the heart, it is out of all experience to have as much dropsical effusion within the chest as in this case. This case may therefore be regarded as an illustration of the occurrence of chronic affection of the kidney following acute tubal nephritis, and also an illustration of double pleurisy produced by renal disease. Her pleurisy has been treated by the application of dry cups to the chest; she has had, in addition, ten grains oı quinine once a day, and pills of iron, aloes, and strychnia.

The second case gives us the following history:

Mrs. ——, aged thirty-three, English, and admitted to the hospital September 22d. Family history good. Patient was healthy until one year ago, when she began to suffer from attacks of dyspnœa, without cough, which were probably asthmatic in character Vomiting and œdema of lower extremities first occurred about six months ago. During the past two weeks she has suffered from some pain in the back; and her urine has been scanty and high-colored. The vision has always been good. Upon admission the patient presented an anæmic appearance, the breath was short, and the appetite poor. Examination of the urine gave s. g. 1010, albumen and casts. Physical examination of chest negative.

*Sept. 26th.*—Under the influence of diuretics and tincture of iron the patient's urine became more abundant, but giving same results by chemical and microscopical examinations.

*Oct. 28th.*—The patient does not pass much urine; complains of pain in her back and shortness of breath.

Upon physical examination of the chest, the area of cardiac dulness is found to be very much increased, and with this there is a murmur with the first sound of the heart, at the base. This patient now has pericarditis, with considerable effusion of serous fluid into the pericardial sac. There is considerable œdema of the lower extremities, and also considerable fluid in the abdominal cavity. Her face does not show any dropsy, and there is but slight indication of its diffusion by making pressure over the sternum. The question may arise here, is this a case of pericarditis, the inflammation giving rise to the effusion into the pericardial sac; or is it a case of hydros-pericardium, due to the chronic renal affection? There is a slight, but a sufficiently distinct, friction murmur occasionally heard, and this sign, be it ever so slight, indicates pericarditis, with a single exception. Sometimes, when there is a pleurisy of the left side, the action of the heart causes the exterior of the pericardial sac to rub against the pleural surface, causing a friction murmur with the cardiac rhythm, and this is called a cardiac pleural friction murmur. If the murmur were of this kind, it should be heard at the left lateral portion of the pericardium. But the friction murmur is more to the right, nearer to the base; it is superficial in character, being a slight grazing sound.

Taking into account the existence of pericardial effusion, there can be no doubt that the murmur denotes pericarditis. Pleurisy can be excluded because an abrupt line of dulness denotes the boundaries of the distended pericardial sac, good resonance on percussion being found everywhere without these boundaries. A simple enlargement of the heart would not produce the dulness which is here found to extend above the base of the organ. The increased space of dulness in cardiac hypertrophy is downwards and to the left. This patient is not suffering much pain, nor is pain a constant symptom of pericarditis. Pain in this disease is sometimes extreme, and sometimes almost entirely wanting. We have, then, in this case another example of serous inflammation developed in the course of chronic renal disease, belonging among the grave secondary affections.

As regards the measures of treatment addressed to the pericarditis, in this case some soothing applications should be made to the præcordia; a light poultice, or the water dressing covered with oiled muslin, and an abundance of flannel. If the kidneys are found to respond to diuretics, these are indicated for a twofold purpose, as follows: to eliminate urea, and to promote the absorption of the liquid in the pericardial sac. Rigid quietude is to be enforced. There is danger of sudden death by syncope on exertion in cases of pericardial effusion. The condition of the patient will not admit of the employment of the active hydragogues with a view to the absorption of the effused fluid; but if the kidneys do not respond to diuretics, saline cathartics, or perhaps the pulvis purgans, may be advisable. The patient should be well nourished. Digitalis will be likely to be useful, by increasing the power of the heart's action.

The third case illutrates a condition associated with, but probably not dependent upon, the renal disease.

The patient's name is Miss C., aged twenty-two. She was admitted to the hospital on the 2d day of September. Family history good. Since last May she has had more or less œdema of the lower extremities. The dropsy extended up the limbs, appeared on the face, and then about the body. She has had occasional nausea and diarrhœa. Exercise gives rise to palpitation of the heart and want of breath. This patient has a pallid countenance, but this is not as marked as when first admitted. Examination of the urine at the time of admision gave a low specific gravity, with albumen and granular and epithelial casts; subsequently, hyaline casts were found.

September 5th, hydro-peritoneum made its appearance, which has continued and somewhat increased up to this date, October 30th; and at the present time there is, as you see, considerable œdema of the lower extremities. No œdema of the face. The question arises in this case, is this hyro-peritoneum due entirely to the renal disease, or in part to some other cause? Although we have evidences of renal disease, I am quite sure that there is some other affection to account for the hydro-peritoneum. The hydro-peritoneum in renal disease sustains a relation to the dropsy in other parts of the body. But the general dropsy in this case is not an important feature, and this leads us to conclude that the hydro-peritoneum is due to some other disease than the renal disease. It is probably due to disease of the liver. But the expiration of my hour prevents further consideration of the case.

